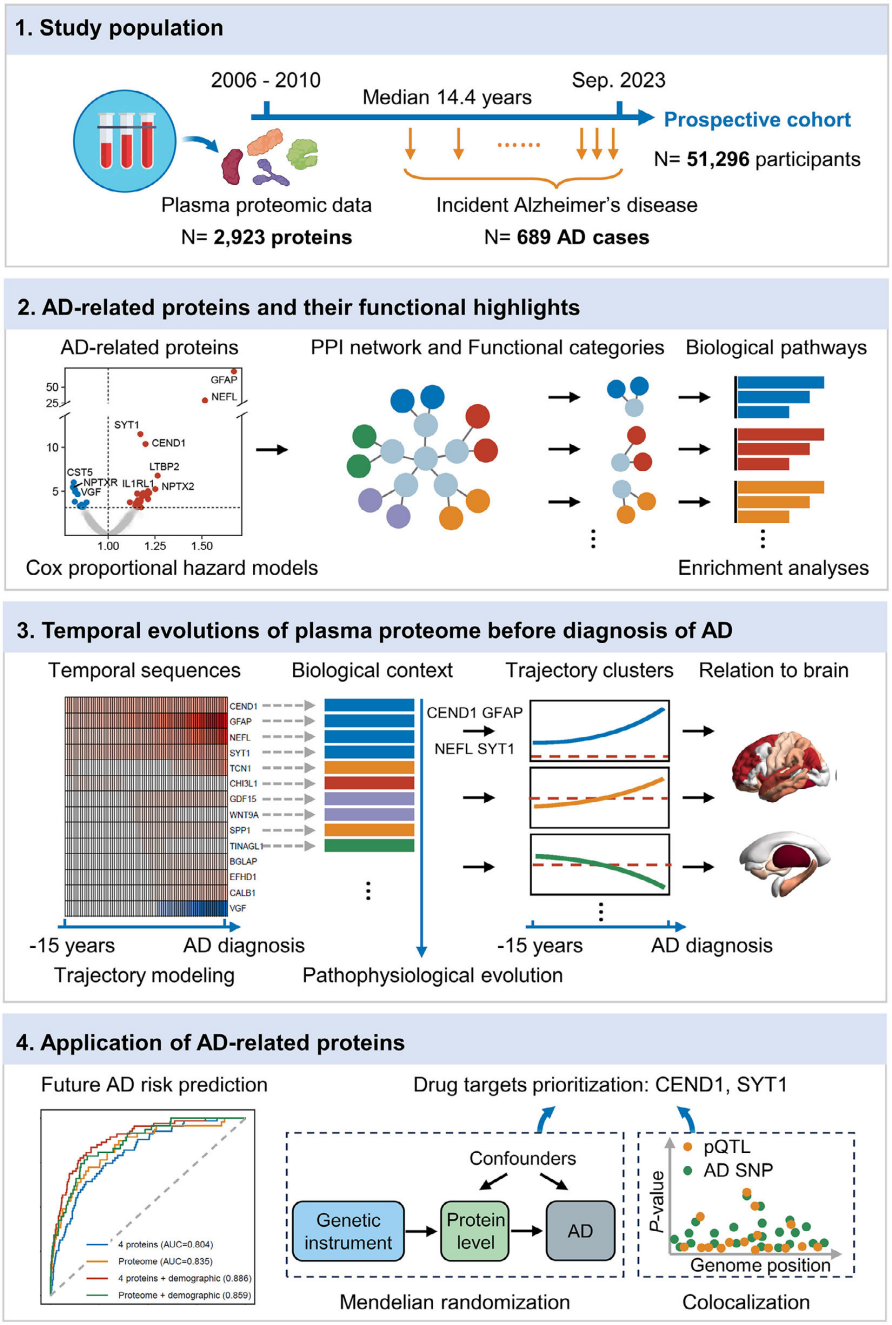# Large‐scale Proteomic Analyses of Incident Alzheimer's Disease Reveal New Pathophysiological Insights and Biomarkers

**DOI:** 10.1002/alz.085077

**Published:** 2025-01-09

**Authors:** Yi Zhang, Yu Guo, JIA YOU, Yu He, Jin‐Tai Yu

**Affiliations:** ^1^ Huashan hospital, Fudan University, Shanghai China; ^2^ Huashan Hospital, Fudan University, Shanghai China; ^3^ Fudan University, Shanghai China; ^4^ HuaShan hospital, Fudan University, Shanghai China; ^5^ Huashan Hospital, Fudan University, Shanghai, Shanghai China; ^6^ National Center for Neurological Disorders, Shanghai China

## Abstract

**Background:**

The pathophysiological evolutions involved in early‐stage Alzheimer’s disease (AD) are not well understood.

**Methods:**

We used data of 2,923 plasma proteins from 51,296 non‐demented middle‐aged adults in the UK Biobank with 15 years of follow‐up. We first identified AD‐associated proteins using Cox proportional hazard models and then delineated their temporal sequences to characterize the pathophysiological evolution. The predictive value and potential as drug targets for proteins were finally estimated.

**Results:**

We identified 48 AD‐related proteins, with CEND1, GFAP, NEFL, and SYT1 being top hits in both near‐term (HR:1.15‐1.77; P:9.11×10^‐65^‐2.78×10^‐6^) and long‐term AD risk (HR:1.20‐1.54; P:2.43×10^‐21^‐3.95×10^‐6^). In trajectory modeling, these four proteins increased 15 years before AD diagnosis and progressively escalated, indicating early and sustained dysfunction in synapse and neurons. Proteins related to extracellular matrix organization, apoptosis, innate immunity, coagulation, and lipid homeostasis also showed early disturbances, followed by malfunctions in metabolism, adaptive immunity, and final synaptic and neuronal loss. We uncovered non‐linear alterations in human plasma proteome with AD progression, and proteins with escalating abnormalities showed stronger correlations with AD signature brain measures. Combining CEND1, GFAP, NEFL, and SYT1 with basic demographic data generated desirable predictions for 10‐year (AUC=0.901) and over‐10‐year AD (AUC=0.864), which were comparable to full model with proteomic and demographic data. In addition, CEND1 and SYT1 were potentially novel drug targets, as supported by Mendelian randomization (P<0.001) and colocalization analyses (Posterior Probability>0.8).

**Conclusions:**

Proteins that first reached defined abnormality and progressively escalated were good predictive markers and potential treatment targets. Our findings highlight the importance of exploring the pathophysiological evolutions in early stages of AD, which is essential for the development of early biomarkers and precision therapeutics.